# Singular Case of Monstrosity

**Published:** 1850-07

**Authors:** J. W. B. Garrett

**Affiliations:** Macon, Tennessee


### WESTERN JOURNAL

OF

### MEDICINE AND SURGERY.

JULY, 1850.

### Art. I.—*Singular Case of Monstrosity.* By J. W. B. Garrett, M. D., of Macon, Tennessee.

I had recently the privilege of observing, with a brother member of the medical fraternity, one of the most remarkable and monstrous cases of deformity ever yet witnessed in the human species; and presuming that it will be somewhat interesting to many of the readers of the Journal, I have assumed the task of giving you an account of the observations of myself and the gentleman before mentioned, in this case.

Dr. Parry, the gentleman alluded to above, was called to attend a negro woman in parturition; and by accident I reached the house of the owner of the woman at the same moment that he did. Dr. P. went almost immediately to the patient's cabin, while I being engaged in transacting some business with Mr. T., the owner of the woman, remained at the house, and was engaged for about an hour. At the expiration of that period Dr. P. came in, and with a face wearing a strange and indescribable expression, he desired Mr. T. and myself to accompany him to the negro cabin. Perceiving from the marked expression of his countenance that something of an extraordinary nature had taken place, I sought to know of him of what nature the circumstance or matter might be. He replied, that the child just delivered of the woman, was so singularly deformed, that he had felt shocked, as well as astonished, by its appearance. And none could have looked at the monster and felt their usual calmness. It was about the size that infants generally are at birth—its weight being seven pounds and eight ounces; and it was, therefore, only in its shape that it appeared so monstrous, and, I may say, terrible. This formation I will now proceed to describe to you, to the best of my ability:

The creature seemed to partake of the form and characteristics of humanity and of the *elephant*. Upon the top of the head, which resembled that of the elephant more than that of man, was a little tuft of hair such as usually covers the head of infants. The ears were placed near the top of the head on each side, and the helix of either one was about three times the ordinary size. The eyes were situated anterior and a little inferior to the ears, and were also on the sides of the head. They were uncommonly small, but in their intimate structure resembled those of man rather than of the elephant. But that which most attracted attention was the form of the nose. The nostrils were placed in their usual position above the mouth; but the fibro-cartilaginous extremity of the organ was about two and three-quarter inches in length, cylindrical in form, about half an inch in diameter at its upper portion, and quarter of an inch at the end, and it hung over the mouth much in the manner that the probosis does over the elephant's. Upon dissection we found this snout or probosis to consist entirely of the fibro-cartilaginous substance that forms the arches and septum of the anterior nares of this organ. No tube or duct could be found in it, nor could we discover any blood-vessels or nerves leading into it. Its color was white with the slightest possible tinge of red; and it was covered by skin like the rest of the face. It was both highly flexible and elastic. The other parts of the nose differed but little from others, except that there was only one turbinated bone on each side of the vomer, and that the ductus ad nasam was much larger.

The mouth was almost in juxtaposition with the nostrils; and what was very remarkable about it was the fact, that in each jaw were twelve well developed teeth; and, most strange of all, the two upper canine or cuspidatus projected obliquely from it to the length of about three-fourths of an inch. All of these teeth were very slightly fixed in their sockets or alveoli, and were held in their places principally by the gums. The tongue was large and short, and the genio-hyo-glossus and lingualis muscles were very thick and strong, as was also the stylo-glossus. The larynx was large, the epiglottis small, the trachea quite large, and the thyroid gland unusually so. The cornua of the os hyoides were very long and wide apart, and the thyroid and cricoid cartilages were blended into one. The sub-lingual and maxillary glands were very large, while the tonsil could not be found. The pharynx and Esophagus were both large—the cuneiform process of the occipital bone, to which the pharynx was attached, being uncommonly long, and the pterygoid processes of the sphenoid bone being very short—which made the sphenoidal cell below the sella turcica a part of the pharynx. The upper maxillary and temporal bones were united together, not by the zygoma and the posterior inferior angle of the malar bone, for there was no zygomatic arch, but by a suture formed by the joining of the posterior edge of the malar portion of the one with the anterior edge of the other, where it generally unites with the posterior edge of the greater ring of the sphenoid bone. The eye was placed near this suture, and consequently as much on the side as in front. Contrary to what is generally, and I may say invariably, the case, the styloid and mastoid processes of the temporal bone were completely formed—the styloid being, however, very short, while the mastoid was quite the opposite. There was no sagittal suture in the frontal bone, which was very low and receding. The coronal and lamdoidal sutures however, allowed the bones forming them to lap far over each other. There were only two pieces of the occipital bone, instead of four; and the posterior portion, extending from the lamdoidal suture to the great occipital foramen, with the posterior portions of the parietal bones, extended far back, so that the back part of the head was at least an inch longer or more prominent than is generally the case; and, notwithstanding this prominence, the trapezius muscles and the ligamentum nuchæ of the neck were extraordinarily thick. Of the cerebrum there was a very small quantity, while the cerebellum was about four times as large—there being but six drachms of the first, and two ounces seven drachms and one scruple of the last. The greatest breadth of the skull, from before backwards, was three and three-quarter inches, and across two and one-eighth; and the greatest depth, from the great occipital foramen to the top of the skull in a vertical line, was one and seven-eighth inches. The arteries and nerves ramified through the brain in a manner as peculiar and eccentric as might be expected of the other singular parts of the monstrous creature; but I will not so far encroach on your time and politeness, as to enter into details, farther than to say that the cerebrum seemed almost made up of them, while the cerebellum was comparatively without either.

Of the viscera of the thorax and abdomen, what I have to say shall be said as briefly as possible. In the first place I have to mention the remarkable fact that there was not in this being the slightest vestige of a thymus gland. The lungs were of the ordinary size, shape, and appearance, and the bronchi communicated with them in the usual manner; but the pulmonary veins, instead of communicating with the left auricle of the heart, passed directly to the aorta just below the point where it gave off the carotid and subclavian arteries. The left ventricle was very small, and had a wrinkled and granulated appearance, which gave it the appearance of having been crisped by fire. There was but little remarkable about the intestines, except that the stomach was astonishingly large, and much more globular in shape than is commonly the case. The spleen was also much longer and thicker, and the liver smaller. The glandulæ renales of the kidneys were also quite large, while near them were the testicles, (for it was a male,) remaining in the cremaster muscle. Upon a close examination it appeared that the gubernaculum had been detached from the testes, as its remains were found in the scrotum. Instead of an epididymis each testicle had a small tubercle on one end, from which proceeded the vas deferens. The peritoneum was remarkably voluminous, forming besides the greater and lesser omenta and mesentery, a number of smaller folds of the same character. It was, also, reflected completely around the pancreas and duodenum. The great omentum was filled with adeps of a transparent color, and when punctured emitted the fluid in a continuous stream. The penis was not much more than half an inch in length, but admitted of considerable elongation when stretched. It seemed to have its main body enclosed in a kind of sac, somewhat after the manner of the horse, and from this side, the penis could be extended.

The other portions of the child, (with some few exceptions, but not very material ones, which I have neglected to mention because I would not encroach too much on your patience,) were perfectly formed—the legs and arms particularly. The skull, heart, end of the nose, with some other parts have been preserved by Dr. P., and he intends making a present of them to some medical institution.

The labor of the mother, in delivering this monstrosity, was not very difficult. She had borne several children previous to the one in question, and all of them were perfectly formed. During the first stages of her pregnancy in this case, she had been much frightened by seeing for the first time, and very unexpectedly, an *elephant* belonging to a traveling menagerie, which was passing through the country. I leave it to you, sirs, who are better able to decide than myself, to account for this singular malformation. There is only one way in which I can do so; and that is in the inexplicable sympathy said to exist between the mother and fœtus. And yet I am as much in the dark as ever when I have done this; for how the wondrous change seen here can be effected by the sympathy that every one knows must exist to a certain extent between the two, I am at a loss to conjecture. Some physiologists affect wisdom enough to account for all such cases, and to explain the modus operandi in which it is effected, but I acknowledge my apprehension to be so obtuse that I could never discover the gist of their arguments. If you or any of your contributors could throw any light on the subject, no one will derive more pleasure from the explication than your obedient servant.

May 7, 1850.

